# Editorial: Understanding and Overcoming Biases in Judgment and Decision-Making With Real-Life Consequences

**DOI:** 10.3389/fpsyg.2022.917896

**Published:** 2022-05-24

**Authors:** Yasmina Okan, Fernando Blanco, Dafina Petrova, Monica Capra, José C. Perales

**Affiliations:** ^1^Centre for Decision Research, Leeds University Business School, University of Leeds, Leeds, United Kingdom; ^2^Department of Social Psychology, Faculty of Psychology, University of Granada, Granada, Spain; ^3^Cancer Registry of Granada, Andalusian School of Public Health, Granada, Spain; ^4^Instituto de Investigación Biosanitaria ibs. Granada, Spain; ^5^CIBER of Epidemiology and Public Health (CIBERESP), Madrid, Spain; ^6^Department of Economic Sciences, School of Social Science, Policy and Evaluation, Claremont Graduate University, Claremont, CA, United States; ^7^Department of Experimental Psychology, Faculty of Psychology, University of Granada, Granada, Spain; ^8^Mind, Brain and Behavior Research Center (CIMCYC), University of Granada, Granada, Spain

**Keywords:** reasoning, judgment, decision-making, cognitive biases, affective biases, decision support

The study of judgment and decision-making is essential to understand human behavior and to inform policy affecting people's wellbeing in different domains, including health, finance, and the environment. Advances in research on judgment and decision-making over the last decades have helped to document a wide range of cognitive and affective biases that can affect decision-making, uncover the mechanisms underlying such biases, and identify moderating factors. However, a better understanding of the impact of biases on judgments and decisions beyond laboratory settings and ways to prevent negative real-world outcomes is still needed. With the current Research Topic, we aimed to bring together researchers from different fields and traditions to cover recent advances in these areas and bridge gaps between theoretical and applied work. We launched the Research Topic in 2020 as an initiative from the Society for the Advancement of Judgment and Decision-Making Studies (SEJyD), which was founded in Spain in 2014 with the aims of creating a new platform for sharing insights from research in this field, promoting interdisciplinary work, and fostering new international collaborations.

We were pleased to receive a diverse set of contributions, resulting in 14 published articles involving 57 authors from 7 different countries (Australia, Italy, Norway, Sweden, US, UK, and Spain). The contributions include theoretical and applied work reflecting expertise in different areas of psychology (experimental, clinical, and health psychology), health sciences, business management, organizational behavior, and sustainability, among other disciplines. The articles spanned diverse methodologies, including large scale laboratory and field experiments, cross-sectional and longitudinal surveys, and syntheses of previous research. Overall, the contributions can be grouped in four main areas of research, outlined below.

The first line of research investigates basic processes in causal learning and judgments of causal relationships in different contexts. Greenaway and Livesey report a contingency learning experiment where participants were presented with pairings of food items (varying in their a priori likelihood to produce allergic reactions) and allergy episodes. Results revealed that both prior knowledge and contingency information contribute to causal beliefs about foods and allergy, even when people are instructed to ignore prior knowledge. Relatedly, Blanco et al. report three contingency learning experiments investigating judgments about the effectiveness of medical treatments. Results indicated that treatments can be perceived as less effective than they are because patients' judgments are systematically biased by the base rate of symptoms. This tendency was driven by people's tendency to use relative, rather than absolute, measures of effectiveness to assess how well treatments work.

The second line of research focuses on investigating factors moderating biases in different domains and underlying cognitive and affective processes. In the domain of environmental decision-making, Threadgold et al. focus on the “negative footprint illusion”, which refers to people's tendency to incorrectly believe that adding “eco-friendly” items (e.g., environmentally certified houses) to a set of conventional items (e.g., standard houses) reduces the carbon footprint of the combined set of items. Reduced susceptibility to the illusion was associated with actively open-minded thinking across two studies, but not with other reflective thinking dispositions. Relatedly, Muela et al. examine the role of individual differences in domain-general reasoning abilities in the context of problem gambling. Such reasoning abilities were mostly unrelated to sensitivity to gambling biases, suggesting that psychoeducation to improve domain-general reasoning could be insufficient to debias gambling-related beliefs and cognitions. Focusing on emotional and motivational factors underlying gambling-related biases, Philander and Gainsbury found that positive attitudes toward electronic gaming machines correlated with overconfidence in understanding how these machines work. However, a manipulation of the provision of accurate and inaccurate information about how outcomes were determined did not influence attitudes, suggesting that information-based interventions may be insufficient to reduce biases and positive attitudes toward gambling. Finally, Mayiwar and Björklund examined the interplay between psychological distancing and emotions in risky judgment and decision-making. The relationship between fear and risk-taking was found to be negative in the absence of psychological distancing but positive in the presence of distancing. These findings suggest that distancing may help to avoid excessive risk aversion caused by incidental fear.

A third area of research focuses on documenting the impacts of cognitive and affective biases on real-world outcomes and decisions. In the context of consumer behavior, Reutskaja et al. examine how price information affects choices concerning which denomination to use when paying for products (e.g., one €50 bill or five €10 bills) and choice of form of payment (cash vs. debit card). In a series of experiments, consumers exhibited the “price-denomination effect” whereby they anchor on prices when deciding which denomination to use. Using an Ecological Momentary Assessment (EMA) methodology, Colombo et al. investigated the relationship between affective forecasting biases and perceived psychological wellbeing. They found that positively biased forecasting of positive affect (i.e., overestimating positive emotional states) is associated with higher perceived psychological wellbeing and resilience. These results suggest that affective forecasting could function as an adaptive cognitive distortion that boosts people's resilience and mental health. On the other hand, Savioni and Triberti review the role of different cognitive biases in the decision-making and health management of patients with chronic diseases. The authors illustrate how different biases might influence the motivation and agency of patients and propose a process model of how cognitive biases can lead to suboptimal decisions. Garrido et al. studied decision delay–the time patients wait before seeking medical attention after symptoms have started–in acute coronary syndrome patients who survived their cardiac episode. They found that patients who had better knowledge of cardiovascular risk factors reported shorter decision delays, suggesting that knowledge of such factors could play a role in decision-making during an acute cardiac event (i.e., a heart attack). Finally, Sambrook et al. review the role of personal experience with extreme weather events in shaping climate change beliefs and action, as well as the influence of prior beliefs on people's perceptions of climate change impacts. The review highlights the importance of examining processes such as motivated reasoning to understand biases in the interpretation of personal experiences of climate change impacts.

A final line of research focuses on testing the effectiveness of strategies to overcome misconceptions, enhance probabilistic reasoning, and improve risky decision-making. Ferrero et al. examined the effectiveness of refutation texts at debunking misconceptions about education among teacher education students. Through a series of experiments, the authors show that refutation texts reduced teachers' endorsement of misconceptions in the short run but not in the long run. The study shows that, once adopted, misconceptions in education can be highly resistant to change. Focusing on probabilistic reasoning, Cruz et al. developed a graph-based Bayesian network tool representing probabilistic dependency relations between variables. The tool was effective to improve Bayesian reasoning in complex scenarios in which most individuals are prone to committing systematic errors. This tool may be used to improve probabilistic reasoning in risk-sensitive fields such as medical or forensic diagnostics and environmental or economic risk forecasting. Finally, Baltruschat et al. test the effectiveness of a mindfulness-based intervention in reducing risky driving behavior in a group of repeat traffic offenders. Participants who were trained in mindfulness did not show differences in emotional regulation, but showed improved performance in risk situations and had fewer accidents in comparison with control groups

Taken together, the studies in our Research Topic highlight the relevance of research in judgment and decision-making to understand human behavior and inform policies to improve wellbeing. This collection of insightful papers contributes to our understanding of the basic mechanisms underpinning different types of biases, circumstances under which such biases may be more likely to occur, and their real-world impact. The studies reviewed also highlight that more work is needed to understand the different factors that might protect people from biases and identify effective strategies to reduce their potential negative impact, particularly in the long term. We hope that our Research Topic will inspire future efforts along these lines, both in terms of specific issues in need of investigation and in terms of fruitful approaches to tackle these issues, including the combination of different methodologies and disciplines. It is also worth highlighting that many of the contributions endorsed open science practices, including publicly sharing study materials, data, and analysis code, and in some cases pre-registering study protocols. We believe that this sets an excellent example for future work that can help to enhance the transparency, reproducibility, and efficiency of research in this area, and at the same time promote collaborative efforts and quick knowledge transfer relating the important societal challenges addressed.

## Author Contributions

All authors listed have contributed to the conceptualization, writing, reviewing of the work, and approved the submitted version.

## Conflict of Interest

The authors declare that the research was conducted in the absence of any commercial or financial relationships that could be construed as a potential conflict of interest.

## Publisher's Note

All claims expressed in this article are solely those of the authors and do not necessarily represent those of their affiliated organizations, or those of the publisher, the editors and the reviewers. Any product that may be evaluated in this article, or claim that may be made by its manufacturer, is not guaranteed or endorsed by the publisher.

